# Supplementary material for: Function and dynamics of the intrinsically disordered carboxyl terminus of β2 adrenergic receptor
Source: Nat Commun. 2023 Apr 10;14:2005. doi: 10.1038/s41467-023-37233-1 (PMC10085991; doi:10.1038/s41467-023-37233-1)

Supplementary Information

Function and dynamics of the intrinsically disordered carboxyl terminus of β2 adrenergic receptor

Jie Heng[1,2,3,4], Yunfei Hu[5,6], Guillermo Pérez-Hernández[7], Asuka Inoue[8], Jiawei Zhao[4,9], Xiuyan Ma[1], Xiaoou Sun[1], Kouki Kawakami[8], Tatsuya Ikuta[8], Jienv Ding[5,10], Yujie Yang[5], Lujia Zhang[9], Sijia Peng[9], Xiaogang Niu[5], Hongwei Li[5], Ramon Guixà-González[11], Changwen Jin[5], Peter W Hildebrand[7,12,13], Chunlai Chen[2,3,4,9*], Brian K. Kobilka[14*]

Affiliations

[1]School of Medicine, Tsinghua University; Beijing 100084, China

[2]Beijing Advanced Innovation Center for Structural Biology, Tsinghua University; Beijing 100084, China

[3]Beijing Frontier Research Center for Biological Structure, Tsinghua University; Beijing 100084, China

[4]Tsinghua-Peking Joint Center for Life Sciences, Tsinghua University; Beijing 100084, China

[5]Beijing Nuclear Magnetic Resonance Center, College of Chemistry and Molecular Engineering, Peking University; Beijing 100871, China

[6]Innovation Academy for Precision Measurement Science and Technology, Chinese Academy of Science; Wuhan, 430071, China

[7]Charité Universitätsmedizin Berlin, corporate member of Freie Universität Berlin and Humboldt-Universität zu Berlin, Institute of Medical Physics and Biophysics, Charitéplatz 1, Berlin, Germany

[8]Graduate School of Pharmaceutical Sciences, Tohoku University; Sendai, Miyagi 980-8578, Japan

[9]School of Life Sciences, Tsinghua University; Beijing 100084, China

[10]College of Life Sciences, Peking University; Beijing 100871, China

[11]Condensed Matter Theory Group, Paul Scherrer Institute, CH-5232 Villigen PSI, Switzerland

[12]Institute of Medical Physics and Biophysics, University Leipzig; 04107 Leipzig, Germany

[13]Berlin Institute of Health; 10178 Berlin, Germany

[14]Department of Molecular and Cellular Physiology, Stanford University School of Medicine; Stanford, CA 94305, USA

*Correspondence: chunlai@mail.tsinghua.edu.cn (C. C.), kobilka@stanford.edu (B.K.K.)

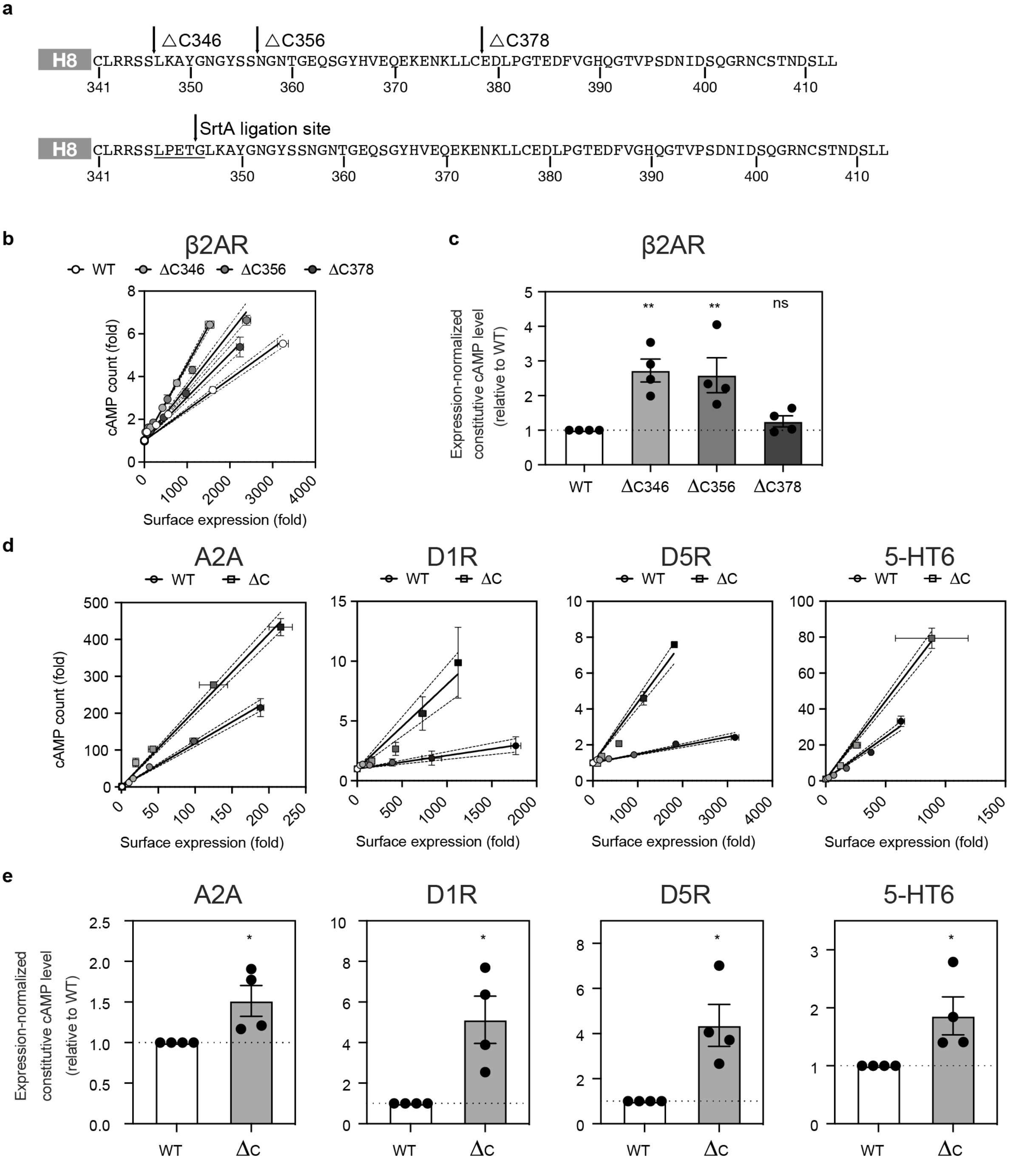

Supplementary Fig. 1 | CTs autoinhibit constitutive receptor activation in five Gs-coupled GPCRs.

a, A diagram shows the sequence of three different ΔCT receptors, and the β_2AR with a SrtA cleavage/ligation site (β_2AR-*s*-CT). **b**, Representative data for surface expression and cAMP level of WT and the three ΔCT β_2AR constructs. HiBiT signal and cAMP GloSensor signal are shown as fold change over mock transfection. β_2AR plasmids were titrated and plotted in the graph with symbols and error bars representing mean and s.e.m., respectively, of 4 technical replicates from a single experiment. Lines and dotted lines indicate linear regression slopes and 95% confidence intervals, respectively. **c**,

Expression-normalized constitutive cAMP level of WT and the three ΔCT β_2AR constructs. The expression-normalized cAMP levels were derived from the slope analysis in **b** and shown as relative value to WT. Bars and error bars indicate mean and s.e.m., respectively, of 4 independent experiments (dots). **, $P < 0.01$; ns, $P > 0.05$ from one-way ANOVA followed by the Dunnett's test with comparison to the WT group. **d**, Representative data for surface expression and cAMP level of 4 additional Gs-coupled receptors (A2A, D1R, D5R, and 5-HT6). GPCR plasmids were titrated and plotted in the graph with symbols and error bars representing mean and s.e.m., respectively, of 2 technical replicates from a single experiment. Lines and dotted lines indicate linear regression slopes and 95% confidence intervals, respectively. **e**, Expression-normalized constitutive cAMP level of WT and the corresponding ΔCT construct. The expression-normalized cAMP levels were derived from the slope analysis in **d** and shown as relative value to WT. Bars and error bars indicate mean and s.e.m., respectively, of 4 independent experiments (dots). *, $P < 0.01$ by the two-tailed *t*-test.

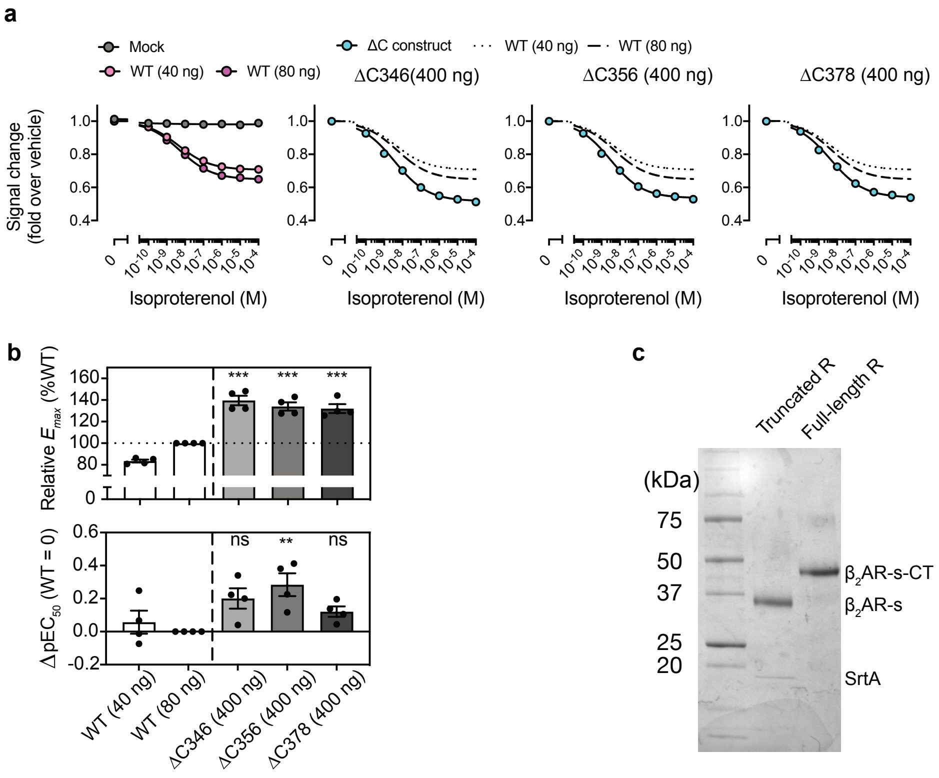

Supplementary Fig. 2 | Agonist-stimulated Gs activity in the WT and CT-truncated β_2AR.

a, Measuring Gαs dissociation from Gβγ stimulated by the full agonist isoproterenol. Bars and error bars represent mean and s.e.m. of 4 independent experiments. Note that, in many data points, error bars are smaller than the size of symbols and thus are not visible. **b**, All ΔCT receptors exhibited a statistically enhanced E_{max} compared to WT receptor. Data are mean ± s.e.m. of 4 independent experiments. Statistical significances between ΔCT and expression-matched WT (ΔC346 vs 80 ng; ΔC356 vs 80 ng; ΔC378 vs 40 ng; also see the Fig. 1a) was performed by one-way ANOVA with the Sidak's multiple comparison test. **, $P < 0.01$; ***, $P < 0.001$; ns, $P > 0.05$. **c**, CT cleavage evaluated by SDS-PAGE in the presence or absence of SrtA.

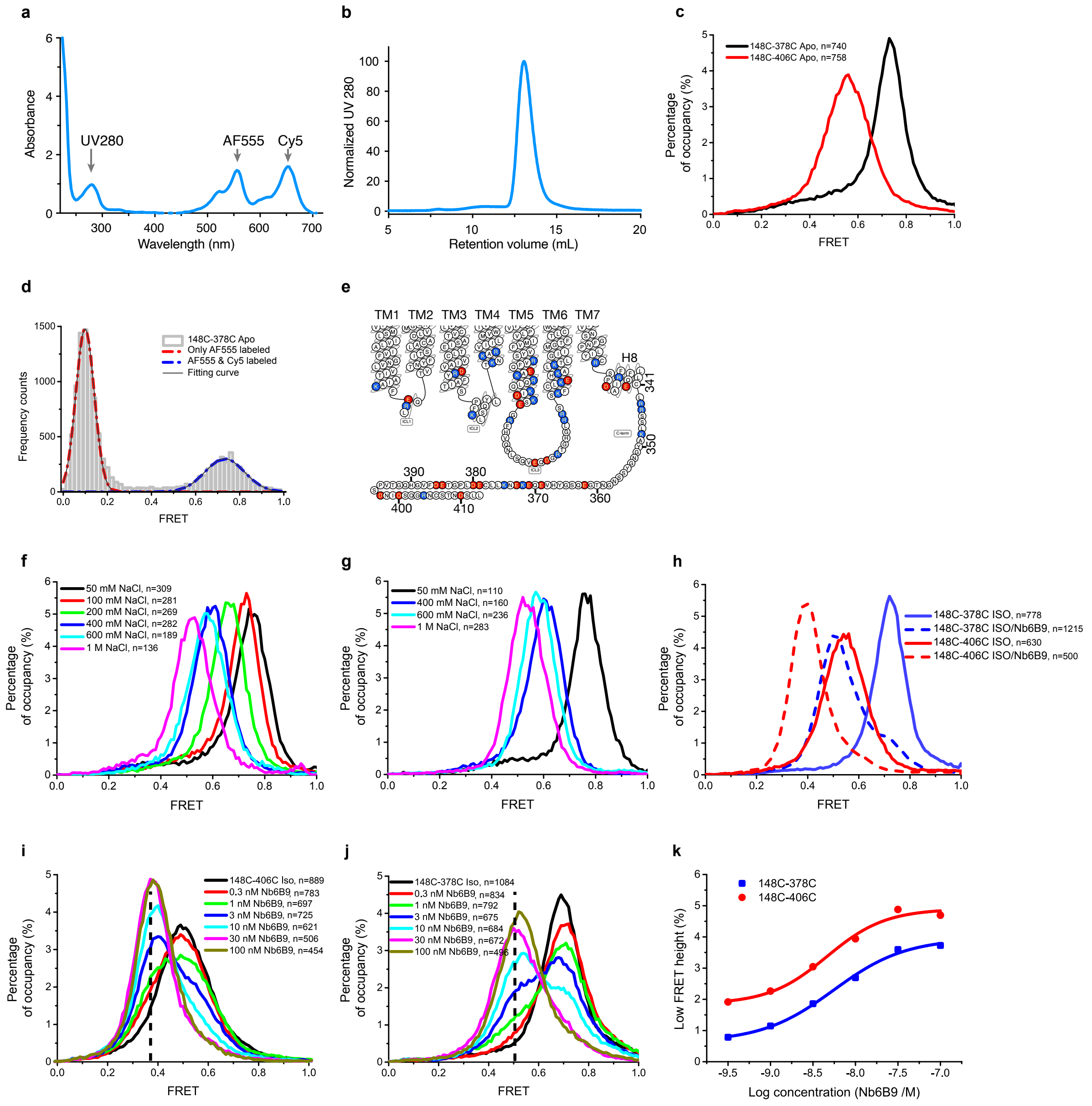

Supplementary Fig. 3 | Receptor preparation and validation of smFRET experiments.

a, The absorbance of labeled β2AR receptor indicates efficient labeling of AF555 and Cy5 dyes. **b**, A representative analytical gel filtration profile of AF555-Cy5 labeled receptor. **c**, A representative FRET histogram of the apo 148C-378C and 148C-406C. Total counts of each FRET sample are normalized to 100%. The FRET center was determined by the Gaussian fitting of peaks. **d**, FRET distribution of freely-diffusing 148C-378C in solution determined by confocal microscopy. The blue peak indicates valid FRET signals that have both AF555 and Cy5 on the receptor, while the red peak indicates only AF555 labeled receptor. The FRET center ~ 0.7 is consistent with the immobilized receptor. **e**, The distribution of charged residues in the intracellular region of receptors. **f**, Receptor 148C-378C was immobilized in a buffer with 50 mM NaCl and high concentrations of NaCl were introduced sequentially

resulting in a shift to a low-FRET state. **g**, Receptor 148C-378C was immobilized in a buffer with 1 M NaCl and low concentrations of NaCl were introduced sequentially resulting in a shift to a high-FRET state. **h**, Nb6B9 causes a leftward shift of the FRET peak in both 148C-378C and 148C-406C. **i**, Dose-dependent effects of Nb6B9 on the FRET distribution of 148C-406C. **j**, Dose-dependent effects of Nb6B9 on the FRET distribution of 148C-378C. **k**, The Nb6B9 concentration-dependent effect on the increment of the low-FRET height. Selected FRET values noted by dotted lines in the presence of different Nb6B9 concentrations from (i,j) were used to calculate EC_{50}.

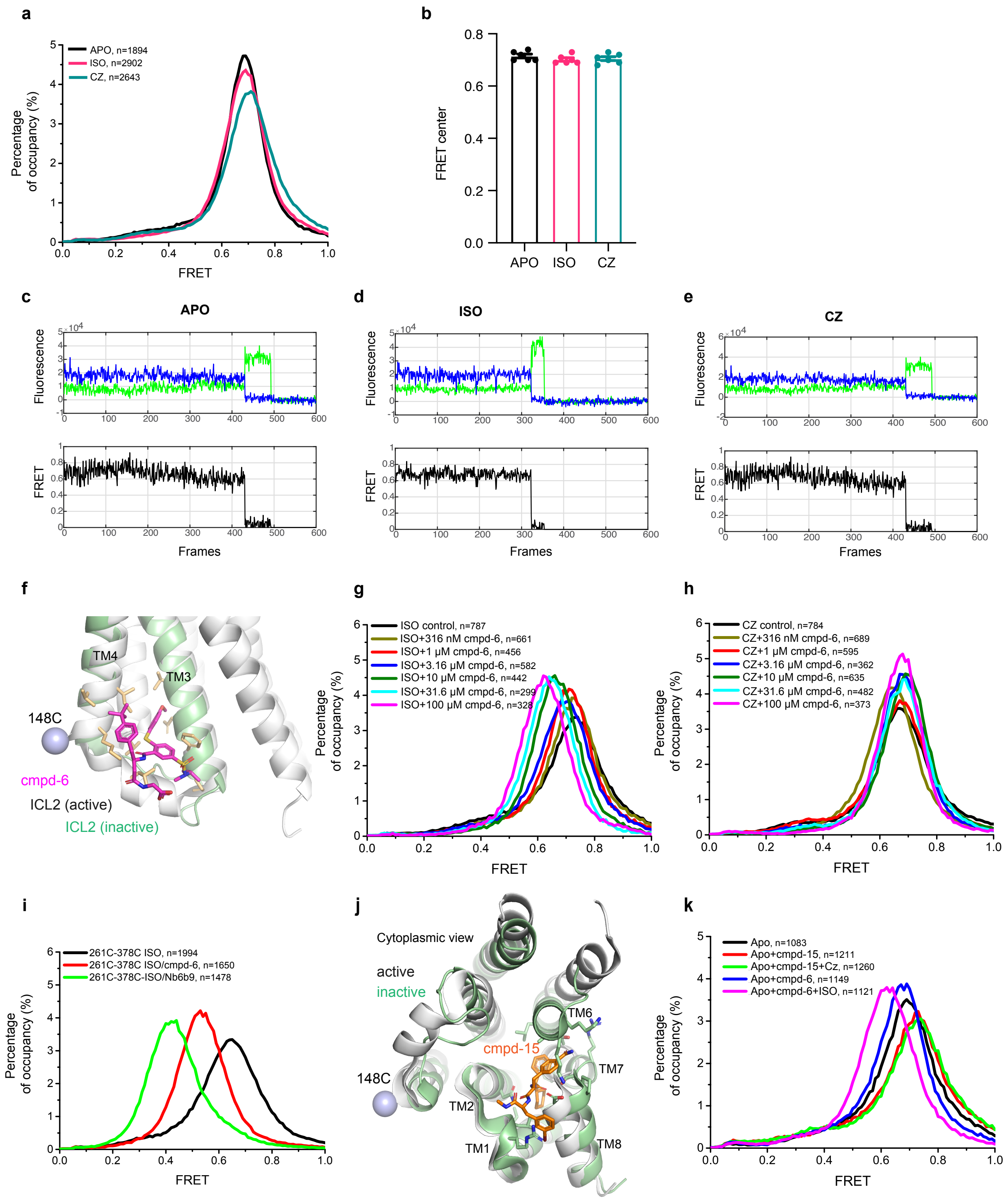

Supplementary Fig. 4 | The effects of orthosteric ligand efficacy on CT dynamics.

a, Representative FRET efficiency histogram of 148C-378C in the Apo, Iso and Cz conditions. **b**, The differences between the Apo and Iso or Cz is not significant. The column and error bars indicate mean and s.e.m. of six independent experiments. Statistical differences between Apo and Iso or Cz were determined by one-way ANOVA with Dunnett's test. **c**, An example single-molecule trace of the Apo

148C-378C. **d**, An example single-molecule trace of the Iso occupied 148C-378C. **e**, An example single-molecule trace of the Cz occupied 148C-378C. The abrupt fluorescent drop at the end of **c**, **d**, and **e** trace represents the photobleaching of fluorophores. **f**, Cmpd-6 binds to a pocket formed by ICL2 and the cytoplasmic ends of TM3 and TM4. Residues that interact with Cmpd-6 are shown as yellow sticks. The fluorophore labeling site 148C is shown as a blue sphere. TM3-ICL2-TM4 of the inactive receptor (PDB: 5X7D), colored in green, are superimposed to the active receptor (PDB: 6N48), colored in grey. **g**, The positive allosteric modulator of the β_2AR, cmpd-6, causes a leftward shift of the FRET distribution for the isoproterenol occupied 148C-378C receptor. **h**, Cmpd-6, doesn't have any effect on the inverse agonist (carazolol) occupied 148C-378C receptor. **i**, Nb6B9, and cmpd-6, in the presence of saturating isoproterenol, cause a leftward shift of FRET in β_2AR labeled at 261C and 378C. **j**, The negative allosteric modulator cmpd-15 binds to a cavity formed by the cytoplasmic ends of the TM1, 2, 6, and 7 as well as ICL1 and helix 8. **k**, The effect of allosteric modulators (20 μM cmpd-6, 20 μM cmpd-15) alone or with orthosteric ligands (100 μM isoproterenol or 1 μM carazolol) on β_2AR labeled at the 148C-378C receptor.

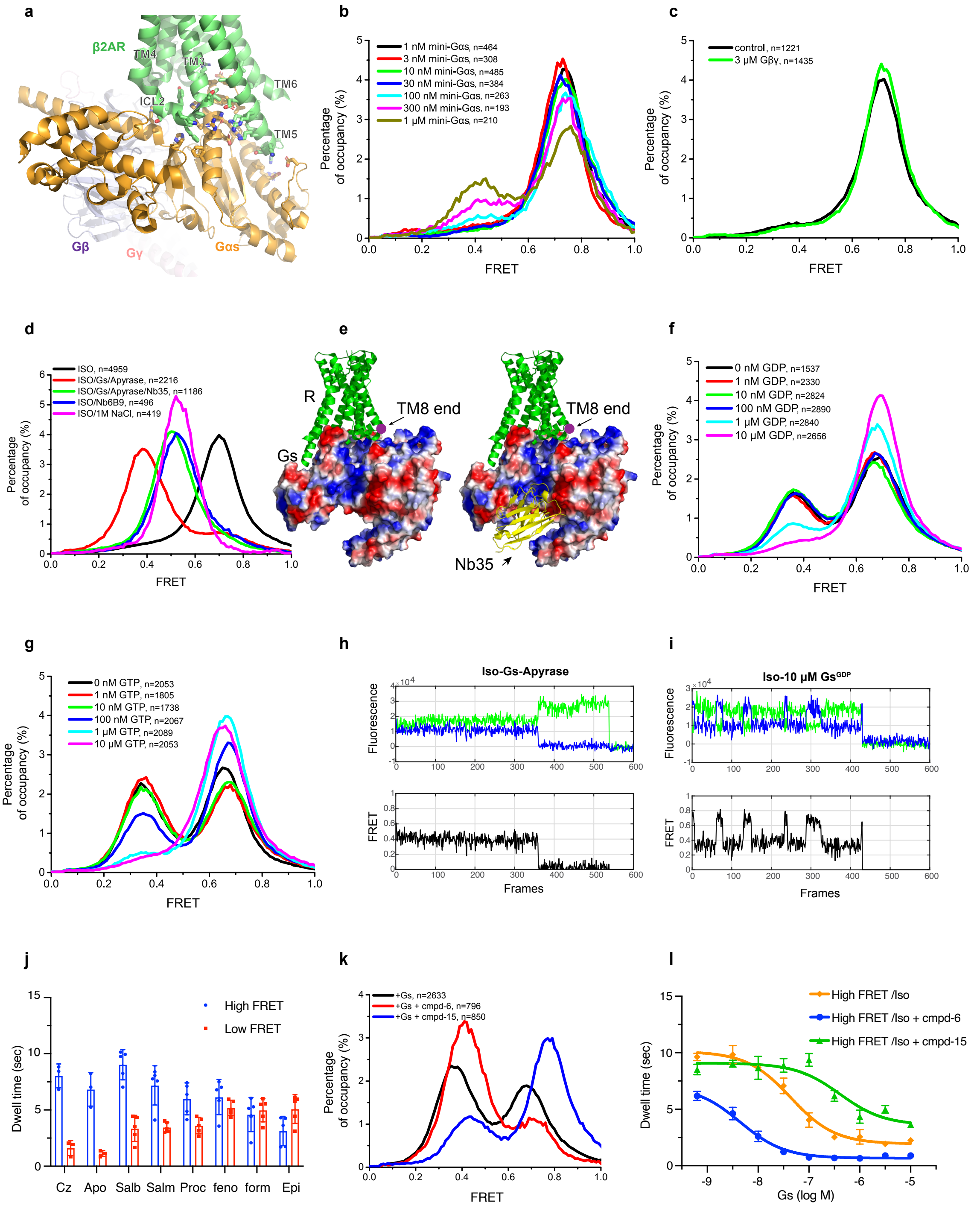

Supplementary Fig. 5 | The effect of Gs and nucleotides on CT dynamics with β$_2$AR 148C-378C receptor.

a, The nucleotide-free β$_2$AR-Gs complex (PDB ID: 3SN6) reveals an extensive interface between Gs and the receptor's cytoplasmic surface. Residues contributing to the interface are shown as sticks. **b**, MiniGαs^{GDP} causes a dose-dependent effect on FRET distribution in the presence of saturating isoproterenol. **c**, Dimeric Gβγ doesn't have any observable influence on the FRET distribution. **d**, The presence of high concentration of sodium chloride, Nb6B9, and nucleotide-free β$_2$AR-Gs complex with or without Nb35 show a leftward shift in the FRET peak. **e**, The electrostatic surface of Gs in the β$_2$AR-

Gs complex. Nb35 binds to a positively charged region of Gas. **f**, The effect of GDP concentration on the FRET distribution of the β_2AR-Gs complex. **g**, The effect of GTP concentration on the FRET distribution of the β_2AR-Gs complex. **h**, An example single-molecule trace of the nucleotide-free, isoproterenol-bound β_2AR-Gs complex. Donor and acceptor fluorescence intensity are shown as green and blue lines, respectively. FRET efficiency is shown as a black line. **i**, An example single-molecule trace of isoproterenol-bound β_2AR in the presence of 10 μM Gs^{GDP}. **j**, Ligands with different efficacy exhibit different abilities to affect the FRET dwell times. The high efficacy ligands result in short high-FRET dwell times. Bars and error bars represent mean and s.e.m. of 3 or 4 independent experiments. **k**, The effect of the positive allosteric modulator (cmpd-6) and negative allosteric modulator (cmpd-15) on the FRET distribution of the isoproterenol-bound β_2AR-Gs^{GDP} complex. Cmpd-6 enhances the formation of a low-FRET population, while cmpd-15 has the opposite effect. Experiments were conducted with 0.5 μM Gs^{GDP} alone or with 20 μM cmpd-6 or 20 μM cmpd-15. **l**, The high-FRET dwell times of the β_2AR CT in the presence of Gs^{GDP} with and without cmpd-6 or cmpd-15. The dots and error bars represent mean and s.e.m., respectively, of three technical replicates.

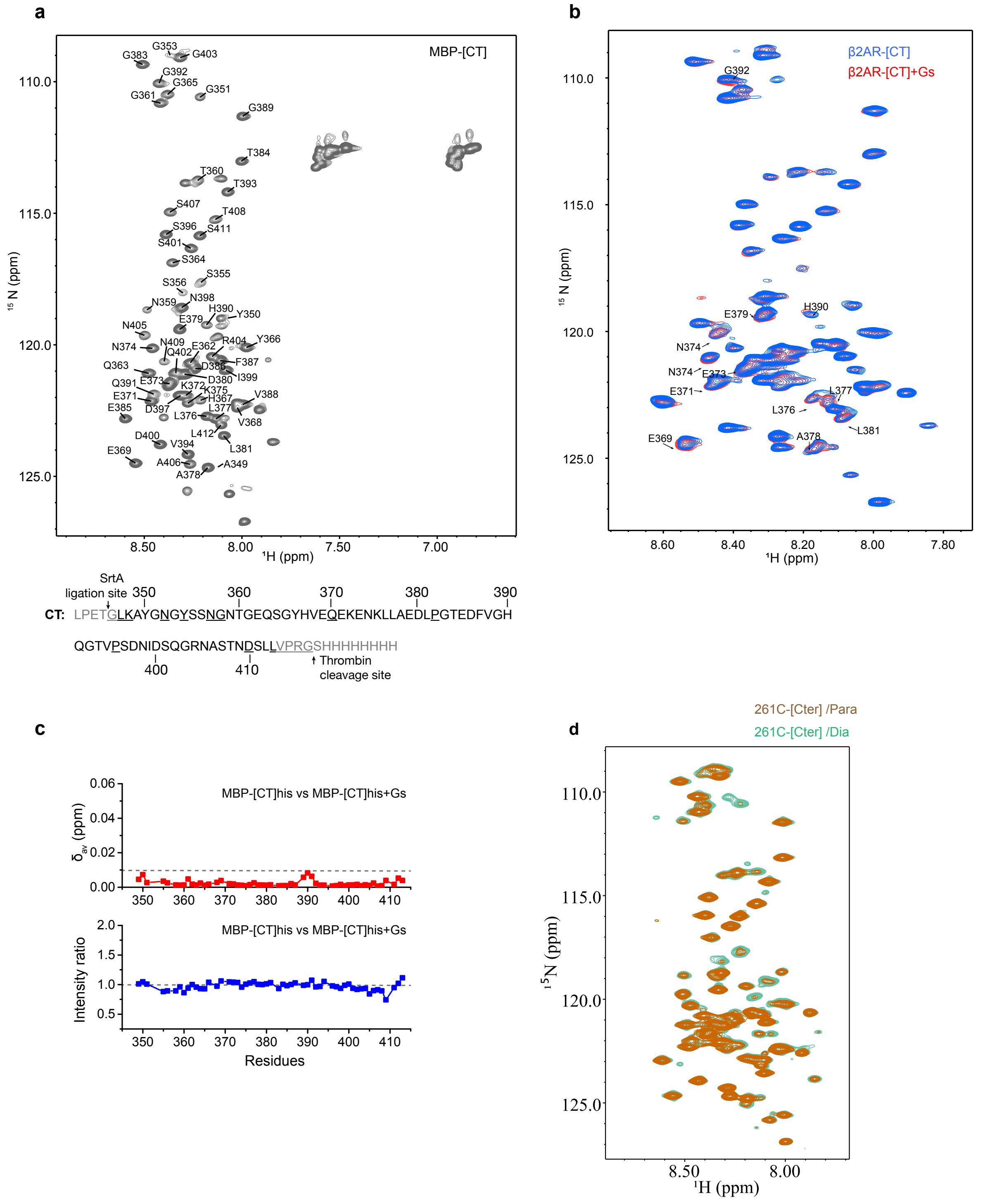

Supplementary Fig. 6 | The NMR studies on ^{15}N labeled β$_2$AR-[CT].

a, The assignment of MBP-[CT]. The underlined residues are the unassigned ones. Note that prolines have no amide group and thus do not have peaks in the HSQC spectra. **b**, The superposed spectra of the ^{1}H-^{15}N HSQC spectra of BI-167107 occupied ^{15}N β$_2$AR-[CT] (blue), and BI-167107 occupied ^{15}N β$_2$AR-[CT] bound to nucleotide-free Gs (red). **c**, The weighted chemical shift changes ($\Delta\delta_{av}$) of ^{15}N MBP-[CT, His] in the absence and presence of Gs. Weighted average changes were calculated as

$\Delta\delta_{av} = ((\Delta\delta_H)^2 + (\Delta\delta_N/5)^2)^{1/2}$. **d**, The superposed ^1H-^{15}N HSQC spectra of BI occupied paramagnetic 261C-TEMPO β_2AR-[Cter] (brown), and diamagnetic 261C-TEMPO β_2AR-[Cter] (green)

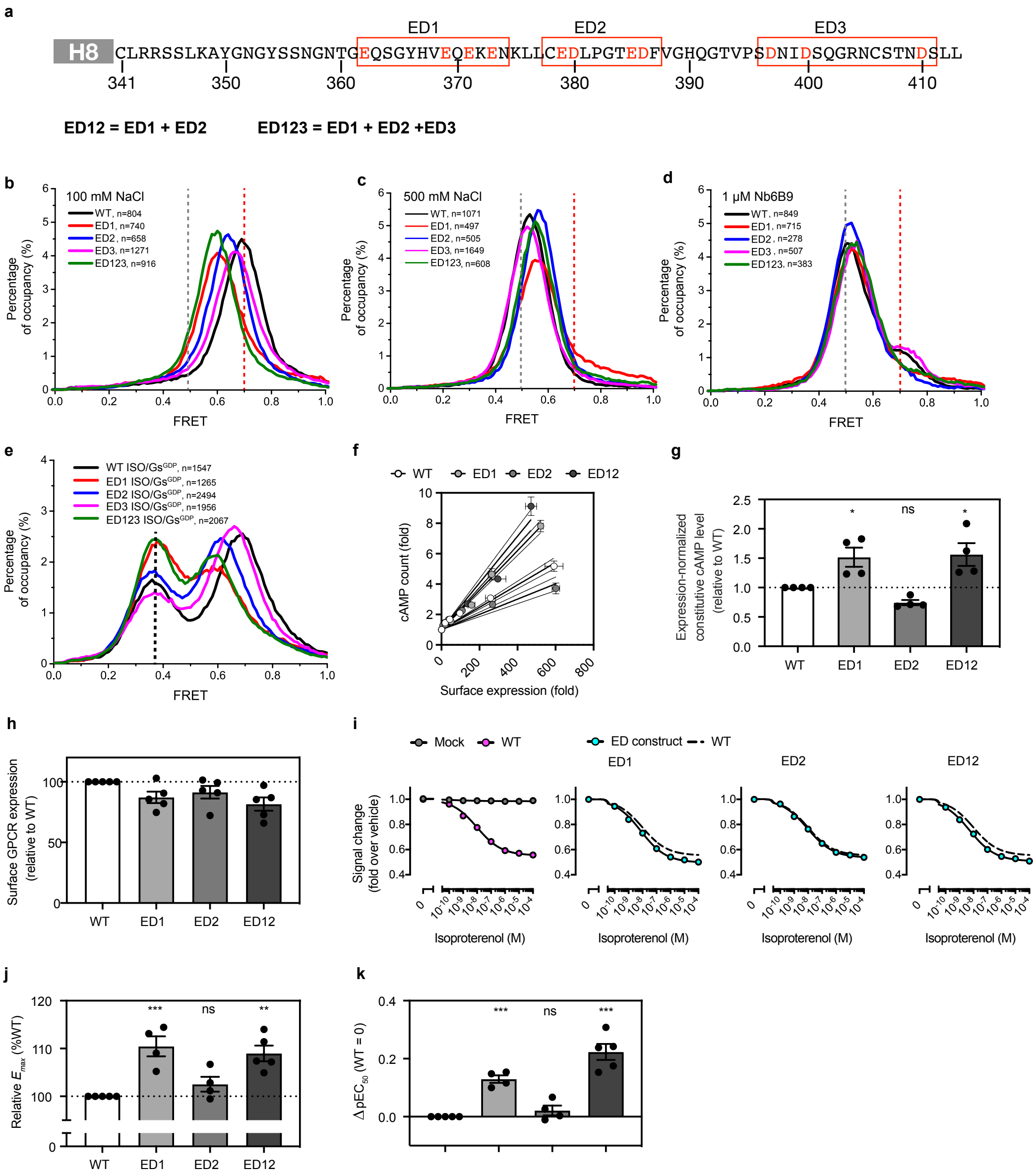

Supplementary Fig. 7 | The collective effect of negatively charged residues in the CT on the smFRET distribution of 148C-378C, G protein coupling, and downstream cAMP signaling.

a, Eleven negatively charged residues are divided into three clusters: ED1, ED2, and ED3. Negatively charged residues in the three clusters were mutated to Ala, and additional mutants were generated by combining selected clusters. **b**, FRET distributions of WT, ED1, ED2, and ED3 of 148C-378C receptor in the presence of 100 mM sodium chloride. **c**, All mutants have similar FRET distributions in the presence of 1 M NaCl. **d**, All mutants have similar FRET distributions in the presence of saturated isoproterenol and 1uM Nb6B9, which can displace the CT when binding to the intracellular surface of the receptor. **e**, ED1 and ED123 enhance the low-FRET population in the presence of Gs^{GDP}, while ED2 and ED3 have little effect. **f**, Representative data for surface expression and cAMP level of WT and the three ED-cluster-mutant β$_2$AR constructs. HiBiT signal and cAMP GloSensor signal are shown as fold change over mock transfection. β$_2$AR plasmids were titrated and plotted in the graph with symbols and

error bars representing mean and s.e.m., respectively, of 4 technical replicates from a single experiment. Lines and dotted lines indicate linear regression slopes and 95% confidence intervals, respectively. **g,** Expression-normalized constitutive cAMP level of WT and the three ED-cluster-mutant β_2AR constructs. The expression-normalized cAMP levels were derived from the slope analysis in **f** and shown as a relative value to WT. Bars and error bars indicate mean and s.e.m., respectively, of 4 independent experiments (dots). *, $P < 0.05$; ns, $P > 0.05$ from one-way ANOVA followed by the Dunnett's test with comparison to the WT group. **h**, Cell surface expression examined by the flow cytometry analysis. ED1, ED2 and E12 expressed at levels comparable to WT β2AR. Dots and error bars represent mean and s.e.m. of 4 or 5 independent experiments. **i**, Measurement of isoproterenol-stimulated Gs dissociation by the NanoBiT-G-protein dissociation assay. Cells expressing the indicated β_2AR constructs were stimulated by full agonist isoproterenol. Data are mean and s.e.m of 4 (for ED1 and ED2) or 5 (for WT and ED12) independent experiments. Note that, in many data points, error bars are smaller than the size of symbols and thus are not visible. **j-k**, Parameters of the Gs dissociation responses in (i). ED1 and ED12 show significantly higher E_{max} and lower EC_{50} values for isoproterenol-stimulated Gs dissociation compared to WT β_2AR. *, $P < 0.05$; ***, $P < 0.001$; ns, $P > 0.05$ from one-way ANOVA followed by the Dunnett's test with comparison to the WT group.

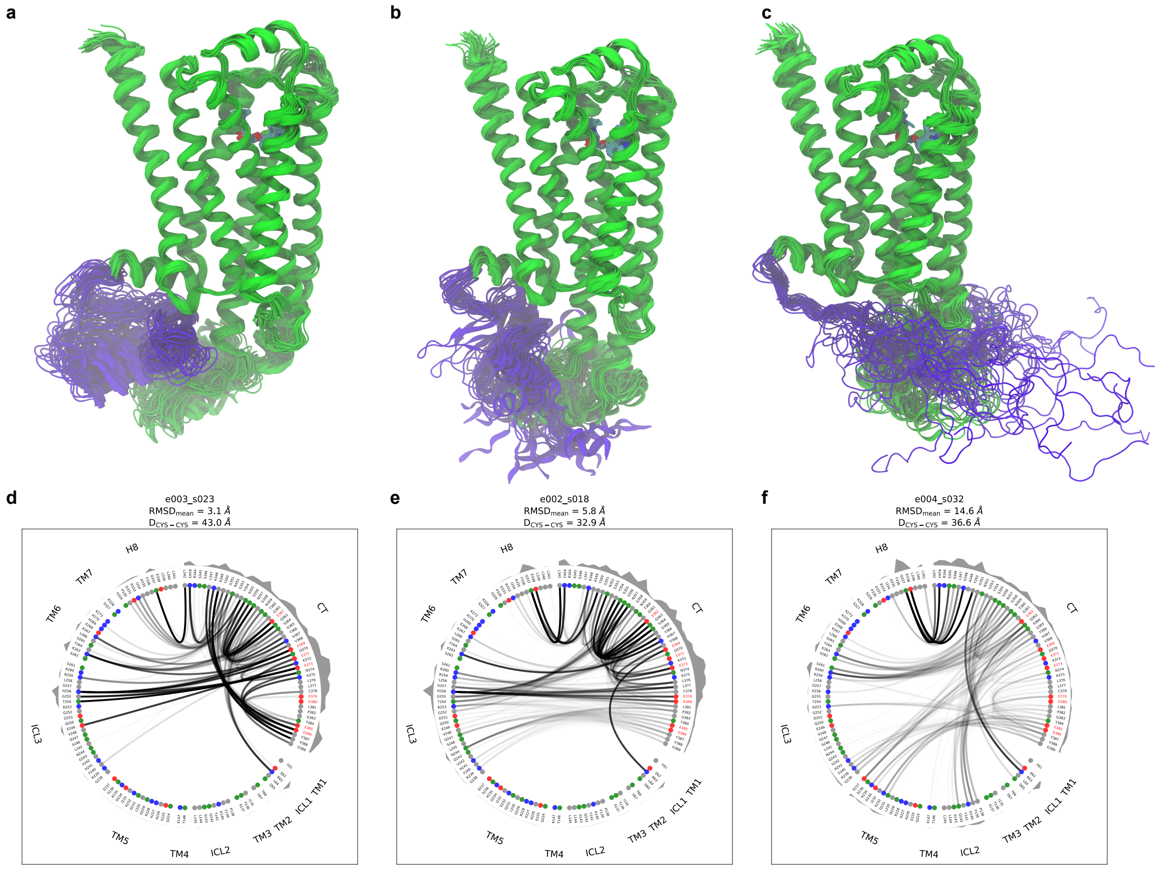

Supplementary Fig. 8 | Variable contacts of the β2AR CT observed in MD simulations.

a-**f**, Three representative trajectories were selected to illustrate different levels of flexibility of the CT in MD simulations of the β_2AR of 200ns length (The CT is shown in purple, whereas the remainder of the receptor is shown in green). The trajectories are shown as overlaid snapshots taken every 2 ns. The companion flareplots, below each receptor figure, illustrate the contacts of the CT. On the outer ring of the flareplot, we show the sum of all the contact frequencies (= curve opacities) for a given residue, indicating the important binding regions. All three trajectories have been made available as interactive mdsrv[1] sessions that can be visualized via the web browser by clicking on the links provided in the zenodo dataset associated with this paper. **a**, Lower flexibility: Example trajectory (#23, CT-RMSD = 3 Å, green curve at the bottom of the third panel of Main Text **Figure 6c**, where the CT remains in a compact conformation during the simulation and with most contacts preserved (flareplot directly below (**d**) with fully opaque lines indicating high occupancy). **b**, Median flexibility: Example trajectory (#18, CT-RMSD = 6 Å, which is close to the median fluctuation value in the last panel of Main Text **Figure 6c**. Parts of the CT, closer to the H8, are more compact, whereas the distal CT residues explore both the inner and outer residues of the ICL3. This is captured by the flareplot below, in (**e**), where we observe a high number of weak contacts between the terminal CT residues and the ICL3 (with pale lines

indicating low occupancy). **c**, Highest flexibility: Example trajectory (#32, CT-RMSD = 15 Å), where the CT explores elongated conformations sampling the outer surface of the TM5-ICL3 region. This is reflected in the flareplot below (**f**), where the elongation is tracked by the loss of CT-CT contacts and very few strong (=fully opaque) interactions between the CT and the receptor core. **d**, Contact flareplot of the CT for trajectory shown in (**a**). **e**, Contact flareplot of the CT for trajectory shown in (**b**). **f**, Contact flareplot of the CT for trajectory shown in (**c**). These trajectories can be 3D visualized via the browser using the link found in the zenodo dataset associated with this paper.

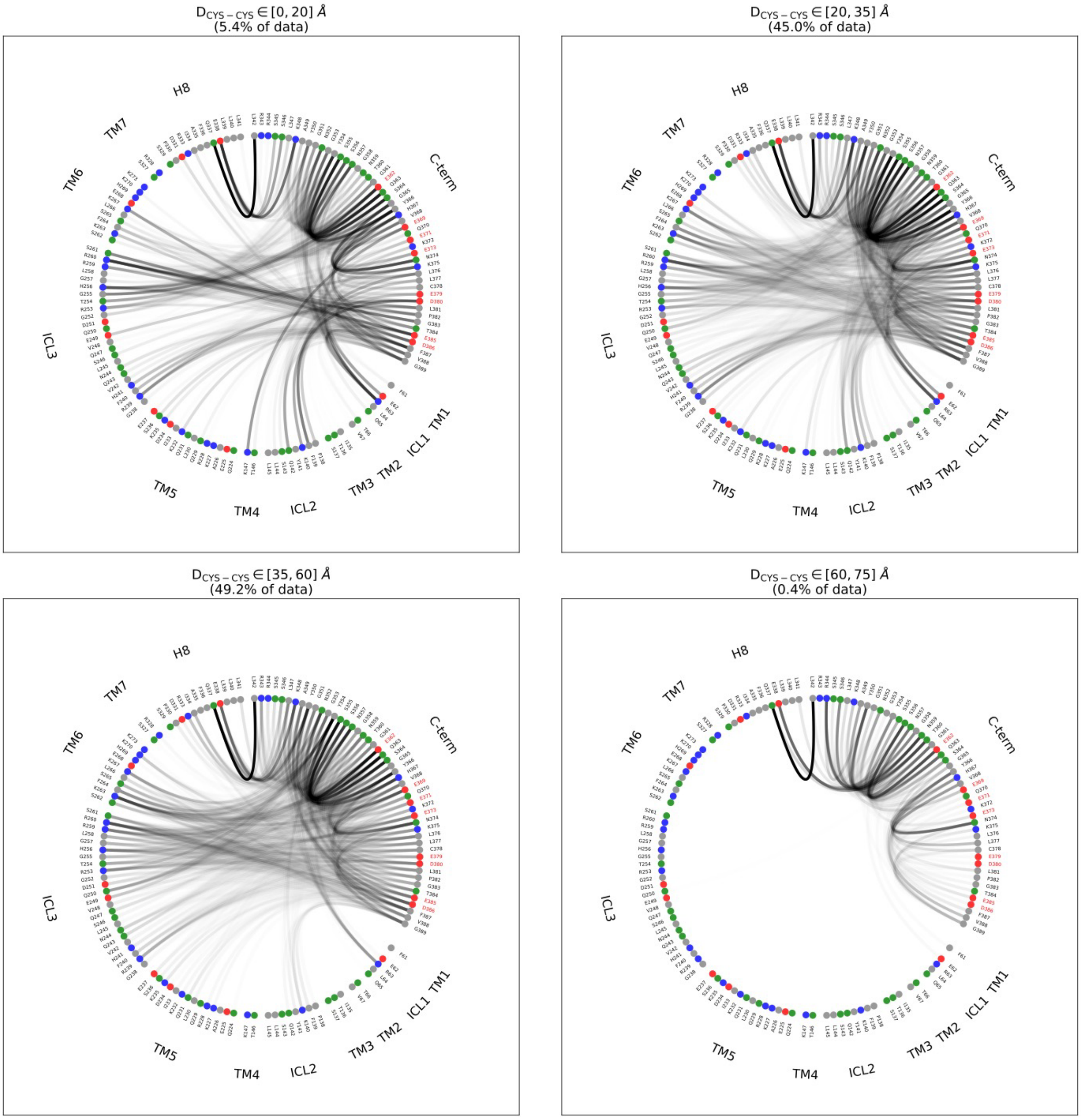

Supplementary Fig. 9 | Variability of contacts of the β$_2$AR CT observed in MD simulations at different CYS-CYS distance ranges.

Each panel shows CT-receptor residue contacts in MD frames falling within 4 specific ranges of C148-C378 distance. In particular, we observe less ICL2-TM4 contacts at higher distances and a complete loss of CT-β$_2$AR contacts at extreme values beyond 60Å (less than 1% of the data).

Supplementary Table 1 | The CT sequence of five Gs coupled GPCRs.

We truncate the receptor CT several residues away from the reported palmitoylation site or the end of Helix 8 (highlighting with underline). The charged residues in receptor CT are colored red and blue. All the CT of five receptors shows a net negative charge.

<table>
<tr><th>Name</th><th>sequence</th></tr>
<tr><td>sp|P07550|ADRB2_HUMAN/322413</td><td>(TM7)NPLIYCRSPDFRIAFQELLCLRRSS<u>LKAYGNGYSSNGNTGEQSGYHVEQEKENKLLCEDLPGTEDFVGHQGTVPSDNIDSQGRNCSTNDSLL</u></td></tr>
<tr><td>sp|P29274|AA2AR_HUMAN/284412</td><td>(TM7)NPFIYAYRIREFRQTFRKIIRSHVLRQQEPFKA<u>AGTSARVLAAHGSDGEQVSLRLNGHPPGVWANGSAPHPERRPNGYALGLVSGGSAQESQGNTGLPDVELLSHELKGVCPEPPGLDDPLAQDGAGVS</u></td></tr>
<tr><td>sp|P21728|DRD1_HUMAN/327446</td><td>(TM7)NPIIYAFNADFRKAFSTLLGCYRLCPATNNAI<u>ETVSINNNGAAMFSSHHEPRGSISKECNLVYLIPHAVGSSEDLKKEEAAGIARPLEKLSPALSVILDYDTDVSLEKIQPITQNGQHPT</u></td></tr>
<tr><td>sp|P21918|DRD5_HUMAN/355477</td><td>(TM7)NPVIYAFNADFQKVFAQLLGCSHFCSRTPVET<u>VNISNELISYNQDIVFHKEIAAAYIHMMPNAVTPGNREVDNDEEEGPFDRMFQIYQTSPDGDPVAESVWELDCEGEISLDKITPFTPNGFH</u></td></tr>
<tr><td>sp|P50406|5HT6R_HUMAN/316440</td><td>(TM7)NPIIYPLFMRDFKRALGRFLPCPRCPRERQAS<u>LASPSLRTSHSGPRPGLSLQQVLPLPLPPDSDSDSDAGSGGSSGLRLTAQLLLPGEATQDPPLPTRAAAAVNFFNIDPAEPELRPHPLGIPTN</u></td></tr>
</table>

Supplementary Table 2 | Average number of contacts of the CT and the β_2AR observed in our MD simulations.

Individual residue-residue contact frequencies have been aggregated to the different regions/fragments of the β_2AR. Frequencies have been computed using a 3.5 Å heavy-atom distance-cutoff.

	**TM1**	**ICL1**	**TM2**	**TM3**	**ICL2**	**TM4**	**TM5**	**ICL3**	**TM6**	**TM7**	**H8**	**CT**
**CT**	0.0	1.0	0.0	0.0	0.8	0.1	0.4	6.2	2.6	0.0	3.5	46.9

Supplementary Table 3 | The most frequent salt bridge in the entire dataset.

From the MD data, the CT residues frequently interact with residues, especially positively charged residues from the ICL3-TM6 region. Due to this region's intrinsically disordered nature, most interactions are non-specific and have a low frequency. Even the most frequent salt bridge in the dataset has a frequency of just 12 %.

Frequency	Contact
0.12	D380@C-term - R260@ICL3
0.12	C378@C-term - R260@ICL3
0.11	L381@C-term - F240@ICL3
0.11	N352@C-term - R63@12.49
0.10	G351@C-term - R63@12.49
0.10	Y354@C-term - F264@6.26
0.10	L376@C-term - K263@6.25
0.10	Y354@C-term - E249@ICL3
0.09	D386@C-term - R260@ICL3
0.09	P382@C-term - R260@ICL3
0.09	Y354@C-term - R63@12.49
0.09	Q363@C-term - F264@6.26
0.09	Y350@C-term - R63@12.49
0.09	E369@C-term - K263@6.25
0.09	G353@C-term - R63@12.49
0.09	Y354@C-term - R253@ICL3
0.09	L376@C-term - S262@6.24
0.09	F387@C-term - R260@ICL3
0.08	Y354@C-term - S262@6.24

Supplementary Table 4 | The average number of contacts of the CT and the β_2AR observed in our MD simulations, at different CYS-CYS distance ranges.

Overall contact frequencies are coarse-grained to the different regions/fragments of β_2AR. Each column has been generated using only the MD frames that fall within the specified C147-C378 distance values, s.t. tendencies can be observed. In particular, we observe the loss of ICL2-TM4 contacts as the distance grows and a complete loss of CT-β_2AR contacts at extreme values beyond 60Å (less than 1% of the data).

	CYS-CYS distance / Å			
	**0-20** [5.4% of data]	**20-35** [45.0% of data]	**35-60** [49.2% of data]	**60-75** [0.4% of data]
**TM1**	0	0	0	0
**ICL1**	1.8	1.4	0.5	0
**TM2**	0	0	0	0
**TM3**	0	0	0	0
**ICL2**	1.6	1.2	0.3	0
**TM4**	0.5	0.1	0	0
**TM5**	0.4	0.3	0.5	0
**ICL3**	6.5	5.7	6.6	0
**TM6**	1.1	2.3	3	0
**TM7**	0	0	0	0
**H8**	3.1	3.5	3.6	2.2
**C-term**	42.6	49.8	44.8	45.2

Supplementary References

1. Tiemann, J. K. S., Guixà-González, R., Hildebrand, P. W. & Rose, A. S. MDsrv: viewing and sharing molecular dynamics simulations on the web. *Nat Methods* 14, 1123–1124 (2017).

The source data in Supplementary Fig. 2C

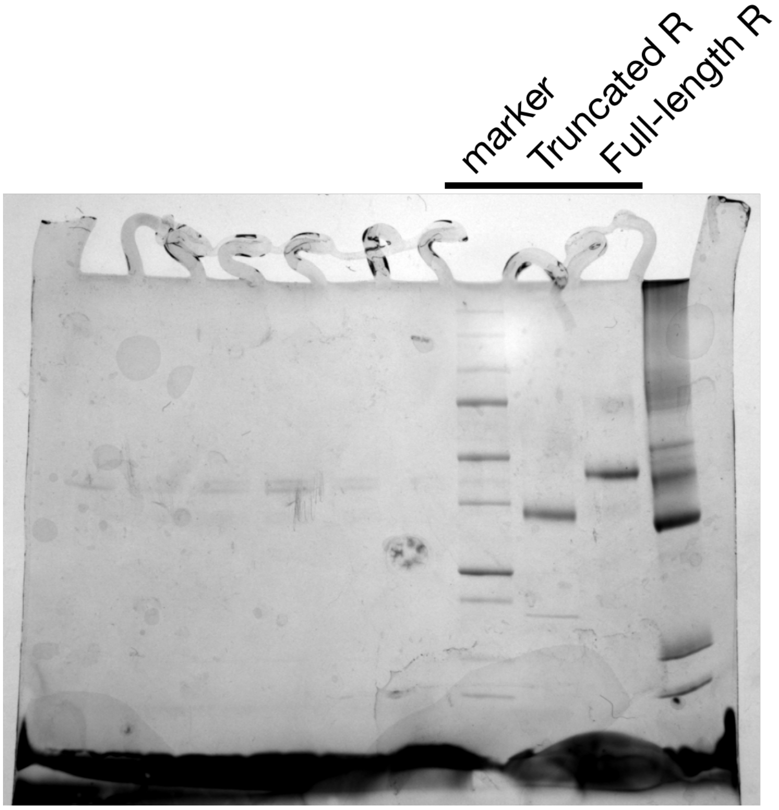

Supplement: Supplementary file 1 — Supplementary Information [file 41467_2023_37233_MOESM1_ESM.pdf]
