# Supplementary material for: Function and dynamics of the intrinsically disordered carboxyl terminus of β2 adrenergic receptor
Source: Nat Commun. 2023 Apr 10;14:2005. doi: 10.1038/s41467-023-37233-1 (PMC10085991; doi:10.1038/s41467-023-37233-1)
Supplement: Supplementary file 3 — Reporting Summary [file 41467_2023_37233_MOESM3_ESM.pdf]

nature portfolio

Corresponding author(s): Chunlai Chen, Brian K. Kobilka

Last updated by author(s): 2023/02/10

Reporting Summary

Nature Portfolio wishes to improve the reproducibility of the work that we publish. This form provides structure for consistency and transparency in reporting. For further information on Nature Portfolio policies, see our Editorial Policies and the Editorial Policy Checklist.

Statistics

For all statistical analyses, confirm that the following items are present in the figure legend, table legend, main text, or Methods section.

n/a	Confirmed	
☐	☒	The exact sample size (*n*) for each experimental group/condition, given as a discrete number and unit of measurement
☐	☒	A statement on whether measurements were taken from distinct samples or whether the same sample was measured repeatedly
☐	☒	The statistical test(s) used AND whether they are one- or two-sided<br>*Only common tests should be described solely by name; describe more complex techniques in the Methods section.*
☒	☐	A description of all covariates tested
☒	☐	A description of any assumptions or corrections, such as tests of normality and adjustment for multiple comparisons
☐	☒	A full description of the statistical parameters including central tendency (e.g. means) or other basic estimates (e.g. regression coefficient) AND variation (e.g. standard deviation) or associated estimates of uncertainty (e.g. confidence intervals)
☐	☒	For null hypothesis testing, the test statistic (e.g. *F*, *t*, *r*) with confidence intervals, effect sizes, degrees of freedom and *P* value noted<br>*Give P values as exact values whenever suitable.*
☒	☐	For Bayesian analysis, information on the choice of priors and Markov chain Monte Carlo settings
☒	☐	For hierarchical and complex designs, identification of the appropriate level for tests and full reporting of outcomes
☒	☐	Estimates of effect sizes (e.g. Cohen's *d*, Pearson's *r*), indicating how they were calculated

Our web collection on statistics for biologists contains articles on many of the points above.

Software and code

Policy information about availability of computer code

Data collection	Fluorescence data were collected using FluorEssence v3.8 (Horiba); Cell Vision software V1.4.0 (Beijing Coolight Technology, ref.67) for data collection of TIRF based fluorescence imaging; NMR data were collected with Bruker TopSpin 3.6, Luminescence data were collected using SoftMax Pro 7.03 (Molecular Devices) and Kaleido 3.0 (PerkinElmer).
Data analysis	We used the following published software and web based analysis tools to analysis our data as referenced in methods: Prism 8.2.1 (GraphPad), Origin9 (OriginLab); PyMol v 2.0 (Schrödinger, LLC), FlowJo software 10.8.1 (FlowJo), ImageJ 1.43 (ref.69), Matlab R2017a, NMRpipe (ref.69), NMRviewJ 9.2.0 (ref.70), HaMMy 4.0 (ref.52), VMD1.9.2(ref.73), SWISS-MODEL server (ref. 74), QUARK (ref.75), HOMOLWAT (ref. 76), CHARMM36m (ref. 77), GROMACS v2018.4 (ref. 78), MDTraj (ref. 83), mdciao (ref. 84)

For manuscripts utilizing custom algorithms or software that are central to the research but not yet described in published literature, software must be made available to editors and reviewers. We strongly encourage code deposition in a community repository (e.g. GitHub). See the Nature Portfolio guidelines for submitting code & software for further information.

Data

Policy information about availability of data

All manuscripts must include a data availability statement. This statement should provide the following information, where applicable:

- Accession codes, unique identifiers, or web links for publicly available datasets
- A description of any restrictions on data availability
- For clinical datasets or third party data, please ensure that the statement adheres to our policy

The backbone 1H, 13C, and 15N chemical shift assignments of MBP-[CT, His] in DDM data generated in this study have been deposited in the BMRB database under accession code 51648.The backbone 1H and 15N assignments of β2AR-[CT] and β2AR-[CT] + Gs in MNG data generated in this study have been deposited in the BMRB database under accession code 51653 and 51656, respectively.
The data showed in the manuscript is uploaded in an source data excel file.
Further information and requests for data and reagents should be directed to and will be fulfilled by the Lead Contact, Brian K. Kobilka (kobilka@stanford.edu).

Human research participants

Policy information about studies involving human research participants and Sex and Gender in Research.

Reporting on sex and gender	N/A
Population characteristics	N/A
Recruitment	N/A
Ethics oversight	N/A

Note that full information on the approval of the study protocol must also be provided in the manuscript.

Field-specific reporting

Please select the one below that is the best fit for your research. If you are not sure, read the appropriate sections before making your selection.

☒ Life sciences ☐ Behavioural & social sciences ☐ Ecological, evolutionary & environmental sciences

For a reference copy of the document with all sections, see nature.com/documents/nr-reporting-summary-flat.pdf

Life sciences study design

All studies must disclose on these points even when the disclosure is negative.

Sample size	Sample size for TIRF based fluorescence imaging was the number of individual molecules. Therefore, in most conditions, approximately, >500 individual molecules were analyzed, which are sufficient for statistical analysis. For TIRF fluorescence imaging, usually 5-15 movies were collected to extract >150 individual single-molecule trajectories in each repeat, which are sufficient to calculate FRET distributions.<br>The fluorescence and luminescence experiments were repeated 3+ times to allow calculation of the mean and standard error of the mean.<br>NMR and SPR measurement was measure with one sample, but three sequential measurements to evaluate the intensity variation of the spectra.
Data exclusions	No data were excluded from the analysis.
Replication	All results were successfully reproduced through at least 2-4 independent attempts.
Randomization	Our samples were not allocated into any experimental groups, therefore randomization is not relevant to our study.
Blinding	During data collection and analysis, there was not involving any group allocation. Therefore, blinding step is not relevant to our study.

Reporting for specific materials, systems and methods

We require information from authors about some types of materials, experimental systems and methods used in many studies. Here, indicate whether each material, system or method listed is relevant to your study. If you are not sure if a list item applies to your research, read the appropriate section before selecting a response.

Materials & experimental systems

n/a	Involved in the study
☐	☒ Antibodies
☐	☒ Eukaryotic cell lines
☒	☐ Palaeontology and archaeology
☒	☐ Animals and other organisms
☒	☐ Clinical data
☒	☐ Dual use research of concern

Methods

n/a	Involved in the study
☒	☐ ChIP-seq
☒	☐ Flow cytometry
☒	☐ MRI-based neuroimaging

Antibodies

Antibodies used	anti-FLAG epitope (DYKDDDDK) tag monoclonal antibody (Clone 1E6, FujiFilm Wako Pure Chemicals; working concentration of 10 µg ml-1); anti-mouse IgG secondary antibody conjugated with Alexa Fluor 647 (Thermo Fisher Scientific; working concentration of 10 µg ml-1).
Validation	We use this commercial antibody by following the instruction form the manufacture.

Eukaryotic cell lines

Policy information about cell lines and Sex and Gender in Research

Cell line source(s)	HEK293 cells deficient for β2AR and β-arrestin1/2 established by and obtained from Asuka Inoue.<br>The Spodoptera frugiperda and Trichoplusia ni insect cells are bought from Expression System.
Authentication	The β2AR and β-arrestin1/2 deficient HEK293 cell line was authenticated by Inoue lab using the PCR and restriction enzyme-based genotyping (ref. 42).
Mycoplasma contamination	All cell lines were tested negative for mycoplasma contamination.
Commonly misidentified lines<br>(See ICLAC register)	No commonly misidentified cell lines were used in this study.
